# A Preliminary Report: The Hippocampus and Surrounding Temporal Cortex of Patients With Schizophrenia Have Impaired Blood-Brain Barrier

**DOI:** 10.3389/fnhum.2022.836980

**Published:** 2022-03-31

**Authors:** Eric L. Goldwaser, Randel L. Swanson, Edgardo J. Arroyo, Venkat Venkataraman, Mary C. Kosciuk, Robert G. Nagele, L. Elliot Hong, Nimish K. Acharya

**Affiliations:** ^1^Department of Psychiatry, Maryland Psychiatric Research Center, University of Maryland School of Medicine, Baltimore, MD, United States; ^2^Center for Neurotrauma, Neurodegeneration, and Restoration, Corporal Michael J. Crescenz VA Medical Center, Philadelphia, PA, United States; ^3^Department of Physical Medicine and Rehabilitation, Perlman School of Medicine, University of Pennsylvania, Philadelphia, PA, United States; ^4^Department of Cell Biology and Neuroscience, Rowan University School of Osteopathic Medicine, Rowan University, Stratford, NJ, United States; ^5^Department of Rehabilitation Medicine, Rowan University School of Osteopathic Medicine, Rowan University, Stratford, NJ, United States; ^6^Department of Geriatrics and Gerontology, New Jersey Institute for Successful Aging, Rowan University School of Osteopathic Medicine, Rowan University, Stratford, NJ, United States

**Keywords:** cerebrovasculature, brain capillary, brain reactive antibodies, autoantibodies, psychosis, serum immunoglobulins

## Abstract

Though hippocampal volume reduction is a pathological hallmark of schizophrenia, the molecular pathway(s) responsible for this degeneration remains unknown. Recent reports have suggested the potential role of impaired blood-brain barrier (BBB) function in schizophrenia pathogenesis. However, direct evidence demonstrating an impaired BBB function is missing. In this preliminary study, we used immunohistochemistry and serum immunoglobulin G (IgG) antibodies to investigate the state of BBB function in formalin-fixed postmortem samples from the hippocampus and surrounding temporal cortex of patients with schizophrenia (*n* = 25) and controls without schizophrenia (*n* = 27) matched for age, sex, and race. Since a functional BBB prevents the extravasation of IgGs, detection of IgGs in the parenchyma is used as direct evidence of BBB breakdown. We also developed a semi-quantitative approach to quantify the extent of IgG leak and therein BBB breach. Analysis of our immunohistochemistry data demonstrated a significantly higher incidence of IgG leak in patients with schizophrenia compared to controls. Further, BBB permeability was significantly higher in advanced-age patients with schizophrenia than both advanced-age controls and middle-aged patients with schizophrenia. Male patients with schizophrenia also demonstrated a significant increase in IgG permeability compared to control males. Interestingly, the extravasated IgGs also demonstrated selective immunoreactivity for neurons. Based on these observations, we suggest that BBB dysfunction and IgG autoantibodies could be two key missing pathoetiological links underwriting schizophrenia hippocampal damage.

## Introduction

Schizophrenia (SZ) is a serious mental illness currently affecting between 0.25–0.64% of the U.S. population and is also one of the top 15 leading causes of disability (Kessler et al., 2005; Gbd 2016 Disease and Injury Incidence and Prevalence Collaborators, 2017). Recent studies conducted in postmortem brain samples of patients with SZ have demonstrated neuropathological changes including a reduction in the hippocampal volume in patients with SZ (Lieberman et al., 2018). Similarly, Savransky et al., have reported microstructural impairments in the fornix, a white matter tract important in hippocampal connectivity, of patients with SZ (Savransky et al., 2017). Furthermore, data from 1963 patients with SZ have revealed a significant reduction in fractional anisotropy, a marker of microstructural integrity, in the white matter areas located at numerous other brain regions (Kelly et al., 2018). These changes in the hippocampus, fornix, and whole-brain and tract-level white matter areas account for much of the cognitive and episodic memory dysfunction in patients with SZ (Kelly et al., 2018; Lieberman et al., 2018; Senova et al., 2020). In spite of these findings, the pathophysiological change(s) and molecular pathway(s) dictating these observations in patients with SZ or SZ-related psychopathology remain unknown.

Multiple recent studies suggest a potential role of cerebrovascular dysfunction in SZ pathogenesis (Pollak et al., 2018). Preclinical studies by Greene et al., suggested a potential decrease of up to 75% in the expression of Claudin-5, a tight junctional protein maintaining blood-brain barrier (BBB) integrity, in SZ and related psychotic disorders of genetic, neurodevelopmental etiology (Greene et al., 2018). Likewise, Berrocal-Izquierdo et al., revealed a higher risk of developing cerebrovascular morbidity in patients with SZ compared to age- and sex-matched controls (Berrocal-Izquierdo et al., 2017). Along these lines, Pinkham et al., reported impaired cerebral blood flow at multiple brain regions of patients with SZ (Pinkham et al., 2011). Recent reports utilizing *in vivo* neuroimaging techniques, Hua et al. (2017) and Najjar et al. (2017) have implicated BBB dysfunction in SZ pathogenesis. Despite these observations, a direct neuropathological study revealing cerebrovascular dysfunction such as increased BBB breakdown in postmortem brain samples from patients with SZ compared to age-matched cognitively normal controls (Ctrls) is missing.

Since most of the vascular components including blood-borne immunoglobulins (IgGs) are normally restricted from entering the brain parenchyma by an intact BBB, presence of IgGs in the brain parenchyma is used as a molecular marker of BBB dysfunction, whereby alternative blood-borne proteins are also found within these leak clouds, like complement C1q, amyloid-beta_42_, and albumin (Clifford et al., 2007; Levin et al., 2010; Nagele et al., 2011; Acharya et al., 2013, 2015, 2017; Goldwaser et al., 2020). Therein, immunohistochemical (IHC) approaches using anti-IgG antibodies are widely employed to assess IgG leak, representing compromised BBB integrity, in formalin-fixed brain tissue samples (Levin et al., 2010; Glass et al., 2017). Currently, IHC approaches to detect IgG leak in formalin-fixed brain samples is primarily qualitative and lack quantitative measure(s) that could be correlated with the extent of IgG leak. To quantify the state of BBB integrity, we developed a semi-quantitative protocol utilizing selective immunoreactivity demonstrated by extravasated endogenous IgGs in the brain parenchyma of formalin-fixed postmortem brain samples from patients with SZ and Ctrl subjects. To estimate IgG leak, we calculated the “IgG leaked area fraction,” a ratio between the area of the hippocampus and surrounding temporal cortex demonstrating IgG immunoreactivity and the area of the entire tissue section. Thus, the IgG leaked area fraction is a direct and semi-quantitative way to estimate the extent of BBB breakdown in formalin-fixed postmortem brain samples. We have used IgG leaked area fraction to compare BBB integrity in the brain sections of patients with SZ and Ctrl subjects.

We observed a significantly higher incidence of IgG leak in the brains of patients with SZ compared to that of Ctrls. Furthermore, advanced-age patients with SZ (51–70 years) demonstrated significantly greater BBB breakdown than middle-aged patients with SZ (30–50 years) and advanced-age (51–70 years) Ctrl subjects. Male patients with SZ also showed a higher incidence of BBB breakdown compared to male Ctrl subjects, which was not the case for females. Interestingly, hippocampal and cortical regions of BBB breakdown both exhibited selective immunolabeling of the neurons and neuropil by extravasated IgGs.

Based on these observations, we believe that in subjects’ with SZ, the temporal cortex surrounding the hippocampus and the hippocampus proper have increased BBB permeability compared to Ctrls. Furthermore, BBB dysfunction is more extensive in the patients with SZ in the advanced-age and male groups.

## Materials and Methods

### Human Brain Tissue

Formalin-fixed postmortem brain samples from the hippocampus and surrounding temporal cortex of SZ (*n* = 25, 35–93 years) and age-matched, neurologically normal, control (Ctrl) (*n* = 27, 29–93 years) subjects were obtained from the NIH NeuroBioBank and Maryland Brain Collection. Patients with SZ and Ctrl subjects’ demographics are listed in Table 1. Exclusion criteria included no evidence of clinical neurodegenerative disease or neurocognitive disorder (such as Alzheimer’s disease, Parkinson’s disease, or vascular dementia) and cause of death not due to intracranial event or processes (such as stroke/hemorrhage, cancer, gunshot wound to the head, traumatic brain injury, etc.) Cause of death is reported in Supplementary Table 1. Three samples were excluded based on cause of death: one due to autopsy revealing “Tau astrogliopathy” and two due to “Subarachnoid hemorrhage.” Including chronic diseases like atherosclerotic cardiovascular disease follows established protocol and was present in equal proportions in both Ctrl and SZ groups, thus mitigating potential biases that favor BBB damage potentially (Glass et al., 2017). Postmortem interval (PMI) of the samples used in the study were ≤25 h (mean [SEM] = 13.7 [0.8] hours). The samples were age, sex, and race frequency-matched (all *p* = 0.2 to 0.9) without significant group differences in PMI (*p* = 0.12) or tissue section size (*p* = 0.2), Table 1. The tissues were processed for routine paraffin embedding, sectioned at 8 μm thickness, and mounted onto Superfrost Plus slides (VWR, Radnor, PA, Cat# 48311-703).

**TABLE 1:** Post-mortem tissue sample demographics and section characteristics.

		Control (*n* = 27)	Schizophrenia (*n* = 25)	Test statistic	*p*-value
Demographics	Age [years] (SD)	51.8 (13.4)	56.9 (14.6)	*t* = −1.3	0.2
	Sex (% male)	58%	56%	χ^2^ = 0.2	0.9
	Race % (C/AA)	20/7	17/8	χ^2^ = 0.2	0.6
Section characteristics	PMI [hours] (SEM)	14.5 (1.0)	11.9 (1.3)	t_50_ = 1.6	0.12
	Average section surface area (μm^2^) (SEM)	1.8⋅10^8^ (1⋅10^7^)	1.6⋅10^8^ (1⋅10^7^)	t_50_ = 1.4	0.17
	sIgG leaked area fraction (μm^2^) (SEM)	8.9⋅10^6^ (2⋅10^6^)	1.5⋅10^7^ (3⋅10^6^)	t_50_ = −1.9	0.06
	Fraction of sIgG leaked area fraction (SEM)	0.05 (.01)	0.11 (.02)	t_50_ = −2.3	0.02
		0.047 (.02)^a^	0.12 (0.2)^a^	*F*_1,48_ = 7.3^a^	0.009^a^

*SD, Standard deviation; C, Caucasian; AA, African American; PMI, Postmortem Interval; SEM, Standard Error of Mean.*

*^a^Estimated means based on univariate analysis with PMI as covariate.*

### Immunohistochemistry

Immunohistochemistry (IHC) was carried out as previously described (Nagele et al., 2011; Acharya et al., 2013). Briefly, the tissue sections were deparaffinized with xylene, rehydrated through a graded series of decreasing concentrations of ethanol and distilled H_2_O and quenched with endogenous peroxidase by treating them with 3% H_2_O_2_ for 15 min. Then, antigen retrieval was carried out by immersing the slides in preheated *Tris*-EDTA buffer in a microwave pressure cooker. Sections were then incubated in 2% Goat blocking serum (Vector Laboratories, CA, Cat# S-1000) in Optimax buffer (BioGenex, San Ramon, CA, United States) for overnight at 4°C. On the following day, the slides were thoroughly rinsed with PBS-T and probed with biotinylated anti-human IgG (Vector Laboratories, CA, Cat# BA-3000, Biotinylated goat anti-human IgG secondary antibody) at 1:250 dilution in 2% goat blocking sera (Vector Laboratories, Cat# S-1000) for 30 min. After thoroughly rinsing with PBS-T, the sections were probed with avidin-biotin complex (ABC, Cat# PK-6100, Vector Laboratories, CA) for 30 min and visualized by treating with 3-3-diaminobenzidine (DAB, Cat# SK-4105, Vector Laboratories, CA). Sections were then lightly counterstained with hematoxylin, dehydrated through increasing concentrations of ethanol, cleared in xylene, and mounted in Permount. Control sections treated with blocking sera or ABC were run alongside the study sections. IHC sections were analyzed and imaged using Nikon FXA microscope equipped with Nikon DXM1200F digital camera, Keyence Digital Microscope (VHX 6000), or Leica Biosystems Aperio CS2.

### Image Analysis

**FIGURE 1:**
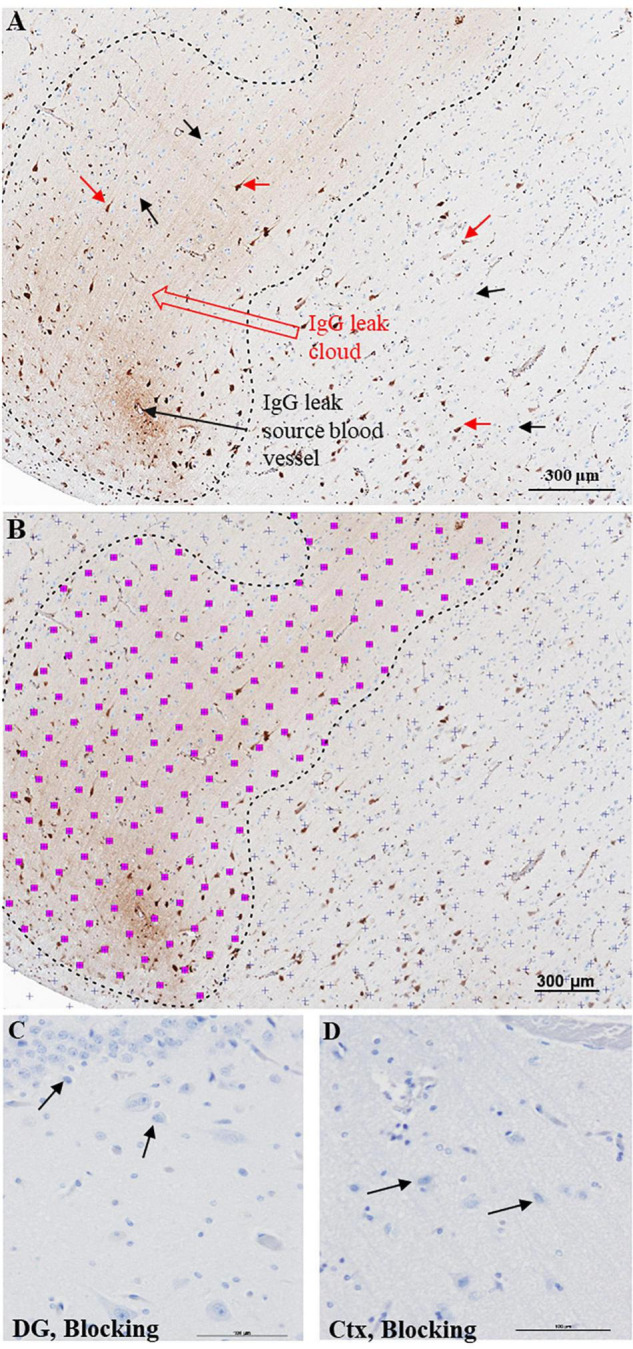
Semi-quantitative approach to quantify BBB breach extent. **(A)** Bright field DAB image of cerebral cortex demonstrating extravasation of IgGs from a leaky blood vessel. The IgG leak forms a characteristic ‘leak cloud’ marked between black dotted lines. Certain pyramidal neurons located within and outside the leak cloud display selective immunoreactivity for IgGs (red arrows), while a larger number of pyramidal neurons fail to show IgG immunoreactivity (black arrows). **(B)** Processed image to measure selective IgG immunoreactivity area using Cavalieri estimator function of stereology software grid system to define non-immunoreactive areas of tissue (purple + marks) and immunoreactive areas (pink squares with purple + marks). This software estimates IgG leak fraction for each section. Scale bar = 300 μm. Hippocampal **(C)** and cortical **(D)** regions from a control slide probed with only 2% blocking serum and ABC reagent. DG = dentate gyrus, Ctx = Temporal cortex, Scale bar = 100 μm.

**FIGURE 2:**
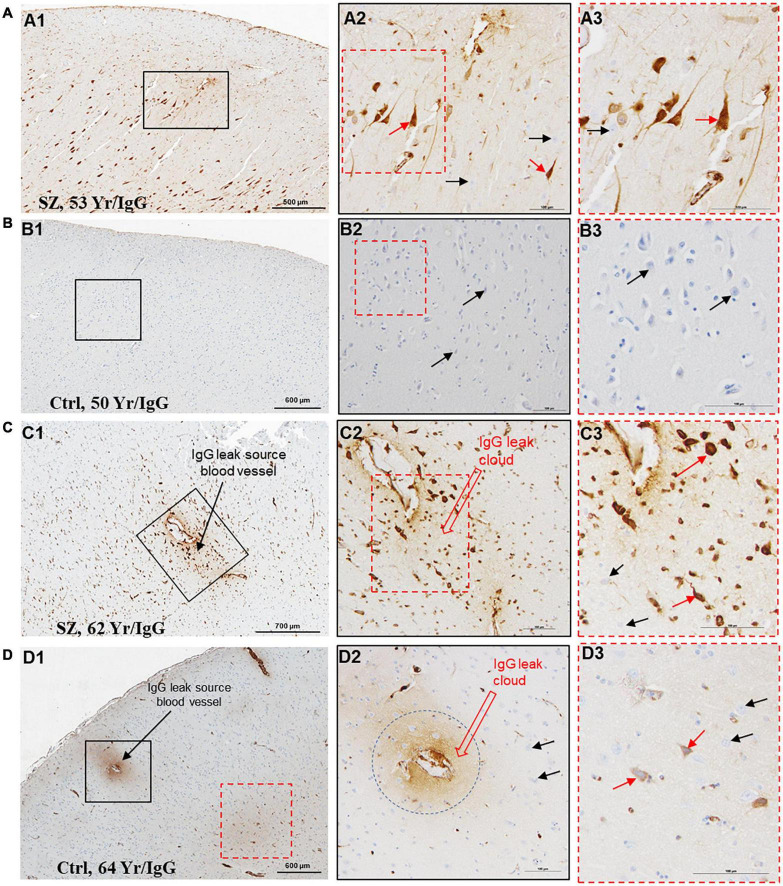
Temporal cortex near hippocampus of SZ patients demonstrates increased BBB permeability and selective interaction between IgGs and pyramidal neurons. **(A–D)** Representative immunohistochemistry images showing the detection of extravasated IgGs (brown color) in parenchyma temporal cortex surrounding hippocampus proper from SZ and Ctrl subjects. These sections were probed with biotinylated anti-human IgG antibodies. As revealed by the brown staining, once in the brain parenchyma, IgGs selectively interact with neuropil and a subset of neurons (red arrows). The temporal cortex demonstrated a greater incidence of IgGs immunoreactivity in SZ patients compared to Ctrls of similar age. The neurons without IgG immunoreactivity are denoted by black arrows. Scale bar (in μm): A = 500 and 100; B = 600 and 100; C = 700 and 100; and D = 600 and 100.

Immunoglobulin Gs are refrained from entering the brain parenchyma by an intact BBB. Therefore, detecting IgG-positive neurons, IgG leak clouds, or IgG immunoreactivity in the neuropil other than in the lumen of the brain vasculature suggests an impaired BBB function. The telltale signs of increased BBB permeability and extravasation of IgGs include (1) focal IgG immunoreactivity around a blood vessel, also referred to as IgG leak source blood vessel, Figures 1A, (2) IgG-positive neurons, Figure 1A, red arrows, or (3) brain parenchyma demonstrating diffuse IgG immunoreactivity, IgG leak cloud, Figure 1A, between the black dotted lines.

Next, we calculated IgG leaked area fraction, the ratio between the area of the hippocampus and surrounding temporal cortex that demonstrated IgG immunoreactivity and the area of the entire tissue section. Since this is critical information necessary for comparing BBB function among the Ctrl and subjects with SZ, we have developed a semi-quantitative protocol using the Cavalieri estimator stereological function in Stereo Investigator software (MBF Bioscience, VT, United States). Similar to previous studies, our semi-quantitative protocol relies on the immunoreactivity demonstrated by the extravasated IgGs with neurons and neuropil (Librizzi et al., 2021; Vila Verde et al., 2021). The Cavalieri estimator method is a proven unbiased stereological method used to calculate areas in tissue sections, and we have adopted it to estimate IgG leaked area fraction or the extent of BBB leak (Cohen, 1992; No authors, 2021).

Blinded investigators used the unbiased grid system of the Cavalieri estimator stereological function of Stereo Investigator software for measuring IgG immunoreactivity in the brain parenchyma of whole slide images (Figure 1B). First, a point grid was laid over the entire slide image section (Figure 1B, purple +). Next, the investigators selected the grid points that hit areas of IgG immunoreactivity (Figure 1B, pink squares with purple + mark). The software calculates the area of a single pink square with purple + based on image magnification. In this study, each pink square with purple + corresponds to the area of 16 pixels or 20 μm^2^. Thus, the area demonstrating IgG leak (immunoreactivity) was accounted according to the following equation: L*eakageArea*_*Section*_ = ∑*Points*_*Leakage*_×*area*_*point*_. [Estimated area of IgG leakage per section (L*eakageArea*_*Section*_) equals the sum of grid points that fall in areas of leakage (∑*Points*_*Leakage*_) times the area represented by a single grid point (×*area*_*point*_)]. Thus, we estimated the *IgG leak area* in each section. Next, we measured the entire area of the tissue section using the Stereo Investigator software. Finally, we calculated the IgG leaked area fraction by calculating the ratio of the area demonstrating IgG immunoreactivity (*IgG leak area in a section*) and the entire tissue section area. In this way, we calculated the extent of BBB breakdown, that is, *IgG leaked area fraction*, for each individual sample. Since the IgG leaked area fraction is proportional to the extent of BBB breakdown, the IgG leaked area fraction represents the extent of BBB leak. We used IgG leaked area fraction to carry out intra- and inter-group comparisons. Negative control samples probed with only blocking sera were shown for the hippocampus (dentate gyrus) and temporal cortex in Figures 1C,D, respectively. Notably, we did not account for the number of IgG immunoreactive neurons in the cortex and hippocampus.

### Statistical Analysis

Subjects’ demographics, clinical characteristics, and tissue parameters were explored for group differences using ANOVA or χ^2^. Differences in the extent of IgG leak between subject groups were compared using raw means and univariate ANOVA covarying for age, sex, and PMI. Multiple comparisons were corrected using false discovery rate (FDR) with the q-value set at <0.05.

## Results

### Blood-Brain Barrier Permeability Is Increased in Patients With Schizophrenia

**FIGURE 3:**
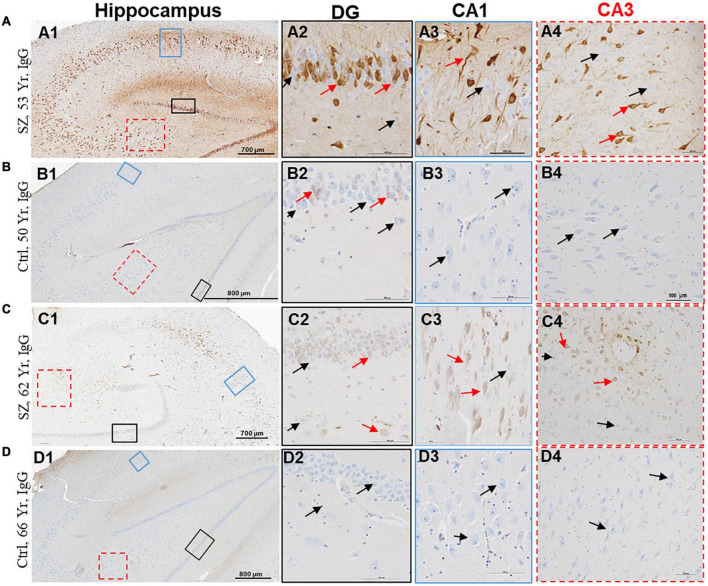
Hippocampus proper of SZ patients demonstrates increased BBB permeability and selective interaction between IgGs and neurons. **(A–D)** Representative immunohistochemistry images showing the detection of extravasated IgGs (brown color) in the parenchyma of hippocampus proper from SZ and Ctrl subjects. Sections were probed with biotinylated anti-human IgG antibodies. Once in the brain parenchyma, IgGs selectively interacted with neuropil and a subset of neurons. A subset of neurons within the DG, CA1, CA2, and CA3 regions of hippocampus demonstrated selective immunoreactivity for IgGs (red arrows). The neurons without IgG immunoreactivity are denoted by black arrows. DG = dentate gyrus; CA = Cornu Ammonis. Scale bar (in μm): A = 700 and 100; B = 800 and 100; C = 700 and 100; and D = 800 and 100.

A functional BBB prevents the extravasation of IgGs from blood vessels into the brain parenchyma. We used IHC techniques to probe formalin-fixed brain samples from patients with SZ and Ctrls with anti-human IgG antibodies (Figures 1–3). Examination of the anti-human IgG IHC sections revealed all three telltale signs of BBB breakdown. First, we have observed blood vessels surrounded by “a brown ring” (Figures 1A, 2C,D), indicating leakage of vascular contents, including IgGs (Figures 1A, 2C,D). When the blood vessel(s) leaking IgGs is accurately identifiable as in Figure 1A, 2C,D, it is termed as *“source blood vessels.”* In brain regions exhibiting BBB breach, all hierarchical types of blood vessels (i.e., arterioles, venules, and capillaries) were found with associated IgG leak clouds. Of these blood vessels, IgG leak clouds were more prevalent in association with arterioles.

The second sign of impaired BBB is the appearance of diffuse brown parenchymal staining (Figures 1A, 2A,C,D, 3A,C,D). The brown staining appearance results from the interaction between the extravasated IgGs and the neuropil comprised of synapses, axons, and dendrites. Although the extravasation of IgGs is responsible for this type of immunoreactivity, in most instances, source blood vessels are not readily identifiable as they could be located either above or below the plane of the brain tissue section.

The third evidence of BBB breakdown is neuronal immunolabeling by extravasated IgGs (Figures 1–3, red arrows, IgG-positive neurons). Interactions between neurons and IgGs are selective in that only specific types of neurons showed IgG immunoreactivity, meanwhile, other neurons under similar exposures (even within the same leak cloud) failed to show IgG immunoreactivity (black arrows, Figures 1–3). This type of selective IgG immunoreactivity is demonstrated by the neurons in the cortex (Figures 1A, 2A1, 2C1,2D1, red arrows) and hippocampus (Figures 3A1,C1, red arrows). It is important to emphasize that our group and others have reported similar types of selective labeling of the neurons in other disease states with BBB breach (Kessler et al., 2005; Hughes et al., 2010; Greene et al., 2018; Lieberman et al., 2018).

**FIGURE 4:**
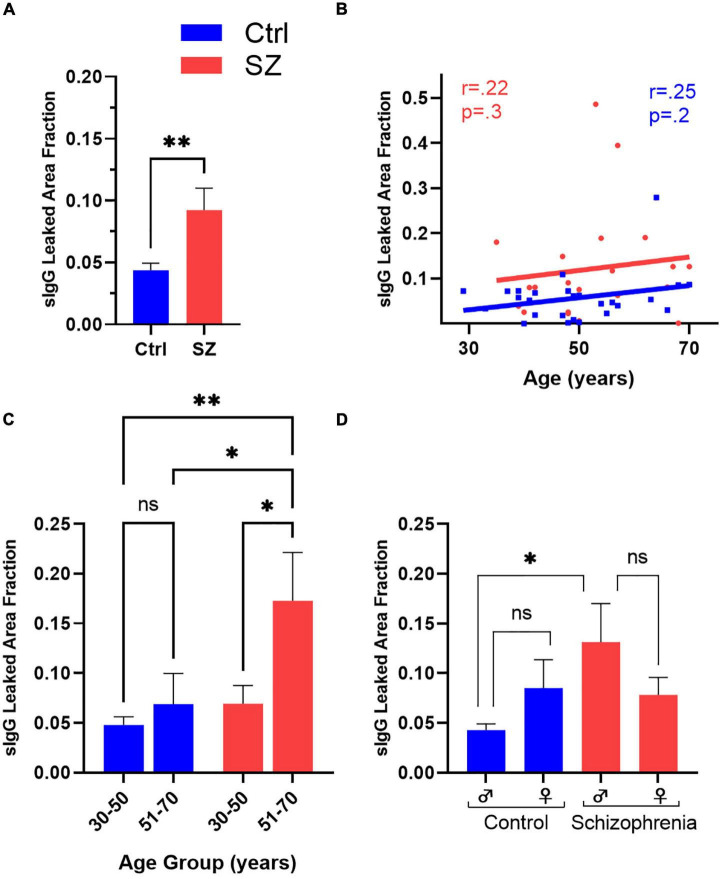
Comparison of the extent of BBB breakdown in the hippocampus and surrounding temporal cortex of the SZ and Ctrl subjects with respect to diagnosis, age, and sex. **(A)** SZ subjects demonstrated a significantly higher hippocampal and temporal cortex IgG leaked area fraction than Ctrls. **(B)** Correlation coefficients of Ctrls and SZ between IgG leaked area fraction and age. Comparison of SZ vs. Ctrls subdivided by middle- and advanced-aged groups **(C)** or gender **(D)** to assess for exploratory inter-and intra-group BBB leakage differences. *p < 0.05, ^**^p < 0.01, with false discovery rate (*q* < 0.05) to correct for multiple comparisons where appropriate.

Using the Cavalieri estimator function of the Stereo Investigator software, we calculated IgG leaked area fraction (Figures 1A,B) and compared the extent of BBB breach between different groups. Since the numerical outcome of the IgG leaked area fraction is directly proportional to BBB breakdown and IgG extravasation, the IgG leaked area fraction is used as an estimation of the extent of BBB leak. We found a significant increase in the extent of hippocampal and temporal cortex BBB leak of patients with SZ compared to Ctrls (t50 = −2.3, *p* = 0.02) (Figure 4A). Linear regression was performed to include age, sex, PMI, and diagnosis as predictors of IgG leak. This model remained significant (F_4,47_ = 3.0, *p* = 0.03) with a significant main diagnosis (*t* = 3.3, *p* = 0.002) effect.

### Aging Increases Blood-Brain Barrier Permeability in Patients With Schizophrenia

Since BBB permeability increases with age, we explored a potential correlation between age and BBB breach in patients with SZ and healthy Ctrl subjects in secondary analyses (Figure 4B). Although the IgG leaked area fraction demonstrated positive trends in BBB permeability with age for both SZ and Ctrl, it failed to reach statistical significance. Next, we compared the average IgG leaked area fraction between the patients with SZ and Ctrl subjects by splitting our sample population into two age groups: middle-aged, 30–50, and advanced-aged, 51–70 years (Figure 4C). We observed a significant increase in BBB permeability in advanced-aged patients with SZ compared to middle-aged SZ patients (*F*_3,39_ = 4.3, *p* = 0.01). On intragroup analysis, although advanced-aged groups of both SZ and Ctrl demonstrated higher IgG leaked area fraction than their respective middle-aged group, only patients with SZ patients attained statistical significance (Figure 4C) (t_39_ = 2.6, p_FDR_ = 0.01). Importantly, the older-aged patients with SZ demonstrated a significantly higher BBB permeability level than Ctrl subjects in both advanced- and middle-aged groups (Figure 4C) (t_39_ = 2.5, p_FDR_ = 0.02 and t_39_ = 3.4, p_FDR_ = 0.001, respectively).

### Exploratory Analysis on Gender and Diagnosis of Schizophrenia With Blood-Brain Barrier Breakdown

Schizophrenia typically develops at younger ages in males compared to females (Marques et al., 2019). Since our sample population comprised a similar number of male and female patients with SZ and Ctrl subjects (Table 1), we ran a univariate analysis to investigate the impact of gender and age on BBB permeability in both patients with SZ and Ctrls in our samples (Figure 4D). IgG leaked area fraction ratio was higher in males with SZ compared to females with SZ, 14.1% vs. 7.8%, respectively (Figure 4D). The intra-group difference in the patients with SZ and Ctrls also failed to reach statistical significance, with a sex x diagnosis interaction term of *F*_1,46_ = 2.2, *p* = 0.1. For exploratory purposes of understanding the impact of gender on BBB permeability between diagnostic groups, univariate analysis showed a significant increase in IgG permeability in male patients with SZ compared to Ctrl males (t_51_ = 2.7, *p* = 0.009), diagnosis x sex interaction (*p* = 0.03) (Figure 4D). No statistical significance was obtained between the female subjects in SZ and Ctrl groups.

## Discussion

This is the first neuropathological study demonstrating increased BBB permeability in the hippocampal and surrounding temporal cortex of patients with SZ. Additionally, we have applied a semi-quantitative approach tallying all evidence of BBB leak together to measure the extent of BBB permeability in postmortem brain samples from patients with SZ and Ctrls.

In this preliminary study, we have observed a significantly higher (roughly double) BBB leak level in patients with SZ than Ctrl subjects. Additionally, patients with SZ in the advanced-age group demonstrated a significantly greater BBB breakdown level than advanced- and middle-aged groups Ctrl subjects. Furthermore, we also observed a greater incidence of BBB permeability in the male patients with SZ than in the male Ctrl subjects. Interestingly, the neurons in the hippocampal and cortical regions of BBB breakdown demonstrated selective immunoreactivity for extravasated IgGs, a potential underpinning for SZ-associated phenotypes.

Our data support a “cerebrovascular dysfunction” theory that associates the initiation and propagation of SZ pathology with BBB breakdown (Najjar et al., 2017; Pollak et al., 2018). Indeed, patients with SZ have a higher risk of developing cerebrovascular morbidity than age and sex-matched controls (Berrocal-Izquierdo et al., 2017), not fully explained by lifestyle/metabolic, substance, or antipsychotic usage (Berrocal-Izquierdo et al., 2018). Likewise, patients with SZ also have impaired cerebral blood flow at multiple brain regions (Pinkham et al., 2011). Hippocampal neuropathologic changes in SZ have been demonstrated with a volumetric reduction on postmortem analysis (Lieberman et al., 2018). Moreover, in live patients with SZ, microstructural impairments in the fornix, a key hippocampal white matter connection, have also been reported as well as inflammatory changes leading to activated microglia (Savransky et al., 2017; Marques et al., 2019). Taken together, the potential role of impaired cerebrovasculature and BBB breakdown in SZ pathogenesis remain both plausible and critically important.

Interestingly, our IHC data presented here reveals selective interactions between the extravasated IgGs and the neurons and neuropil at the site of BBB breakdown. These specific interactions between extravasated IgGs and neurons in patients with SZ are similar to earlier observations in other neurodegenerative and neurological disease states with impaired BBB (Hughes et al., 2010, 2013; Levin et al., 2010; Nagele et al., 2011; Michalak et al., 2012; Acharya et al., 2013, 2015, 2017; Hammer et al., 2014; Goldwaser et al., 2016, 2020). The selective nature of the interaction between leaked IgGs and neurons and neuropil suggests an existence of a subset of IgGs that can bind to its cognate receptors expressed by the neurons and neuropil. For their ability to target these receptors, we refer to this subset of IgGs as brain-reactive IgG autoantibodies (BrABs). Postmortem tissue limits the ability to discern where along the dynamic process of internalization and intracellular digestion a given BrAB is labelling. Temporally, we can conclude that the presence of IgG-reactive neuropil and related structures occurs as sequelae to BBB breakdown, however, it remains unclear where, or when, along the degradation pathway these IgG species belong. Based on our previous studies conducted on *in vitro* systems, selective interactions between purified IgG antibodies against neuronal surface protein receptors promote internalization of extracellular components (Nagele et al., 2011; Goldwaser et al., 2020), akin to autoimmune pathophysiology en route to lysosomal trafficking (Hughes et al., 2010). Results presented herein may further develop and extend this hypothesis to SZ. Future studies aimed at identification of BrABs in sera of patients with SZ and its cognate neuronal surface protein receptor target(s) are essential for the validation and testing of this hypothesis.

The data presented in this study has several limitations. Firstly, the sample size is one apparent constraint that leaves some findings preliminary, powered primarily to observe group differences as the main outcome measure. Second is the lack of availability of the psychopharmacologic and metabolic data for the patients with SZ and Ctrls are confounds unable to be accounted for. Thirdly, in this report, we have presented the state of BBB function in only one brain region due to limited tissue availability. The semi-quantitative method of measuring BBB leak does not separate one evidence of BBB permeability from the other, or differentiate between vessel type or size, thus not accounting for different neuronal cell size in areas of leak. Kept consistently between Ctrls and patients with SZ, however, this limitation was mitigated between groups, but may overestimate absolute values while not changing relative group differences observed. Adhesion molecules and endothelial specific staining protocols were not performed. Similarly, cause of death that includes atherosclerotic cardiovascular disease was found in roughly equal proportions across Ctrl and SZ groups, however, also may likely have its own, independent effect on BBB damage to consider in future studies.

The approach used to compare the extent of BBB breakdown depends on the antigenicity of the brain samples. Despite these limitations, our preliminary report demonstrates novel use of IHC methods to estimate BBB leak in the hippocampal and temporal cortex of patients with SZ. This study lays the foundation for future studies with larger sample-size, multiple brain regions, and robust sample clinical/medication histories. We emphasize that it is almost impossible to find individuals suffering from only one disease, such as SZ. Likewise, Ctrls are also suffering from one or more conditions. Furthermore, many comorbid changes that can influence BBB integrity are associated with aging and chronic disease. For addressing these inherent challenges of using human brain samples, rigorous exclusion and inclusion criteria should be demanded and maintained, especially in cross-study comparisons.

## Conclusion

The data presented in this preliminary study suggests a potential role of impaired cerebrovasculature such as BBB breakdown in SZ pathology. Our data also agrees with earlier reports that suggested a cerebrovascular component as a pathoetiologic mechanism for SZ development.

## Data Availability Statement

The raw data supporting the conclusions of this article will be made available by the authors, without undue reservation.

## Ethics Statement

The studies involving human participants were reviewed and approved by Rowan University School of Osteopathic Medicine. The patients/participants provided their written informed consent to participate in this study.

## Author Contributions

NA, EG, RN, and LH: study conception and design. NA, MK, VV, RS, and EA: experiment and data collection. EG, NA, RS, EA, VV, LH, and RN: data analysis, statistics, and interpretation of results. EG, NA, RN, LH, RS, and VV: draft and manuscript preparation. All authors reviewed the results and approved the final version of the manuscript.

## Conflict of Interest

LH has received or is planning to receive research funding or consulting fees from Mitsubishi, Your Energy Systems LLC, Neuralstem, Taisho, Heptares, Pfizer, Luye Pharma, Sound Pharma, Takeda, and Regeneron. The remaining authors declare that the research was conducted in the absence of any commercial or financial relationships that could be construed as a potential conflict of interest.

## Publisher’s Note

All claims expressed in this article are solely those of the authors and do not necessarily represent those of their affiliated organizations, or those of the publisher, the editors and the reviewers. Any product that may be evaluated in this article, or claim that may be made by its manufacturer, is not guaranteed or endorsed by the publisher.
