# Supplementary material for: A Preliminary Report: The Hippocampus and Surrounding Temporal Cortex of Patients With Schizophrenia Have Impaired Blood-Brain Barrier
Source: Front Hum Neurosci. 2022 Mar 31;16:836980. doi: 10.3389/fnhum.2022.836980 (PMC9008835; doi:10.3389/fnhum.2022.836980)
Supplement: Supplementary file 1 [file Table_1.docx]

**Supplemental Table 1. Individual sample demographics and cause of death.**

| Age | Race | Sex | Group | PMI | Cause of Death | I/E |
| --- | --- | --- | --- | --- | --- | --- |
| 29 | AA | F | Ctrl | 18 | Pulmonary embolism | I |
| 33 | AA | M | Ctrl | 8 | During tonsilectomy | I |
| 37 | AA | F | Ctrl | 24 | Transected aorta | I |
| 39 | C | M | Ctrl | 18 | Undetermined | I |
| 39 | C | M | Ctrl | 16 | Hemopericardium | I |
| 40 | C | F | Ctrl | 13 | Ruptured aorta | I |
| 41 | C | M | Ctrl | 20 | Atherosclerotic cardiovascular disease | I |
| 42 | AA | F | Ctrl | 10 | Occlusive pulmonary thromboembolism | I |
| 42 | AA | M | Ctrl | 9 | Dilated cardiomyopathy | I |
| 47 | C | M | Ctrl | 6 | Atherosclerotic cardiovascular disease | I |
| 47 | C | F | Ctrl | 22 | Acute subarachnoid hemorrhage | E |
| 48 | C | F | Ctrl | 9 | Pulmonary thromboembolism | I |
| 48 | C | F | Ctrl | 13 | Gastric perforation | I |
| 49 | C | M | Ctrl | 23 | Undetermined | I |
| 49 | C | M | Ctrl | 13 | Atherosclerotic cardiovascular disease | I |
| 50 | C | M | Ctrl | 21 | Motor vehicle accident | I |
| 50 | C | M | Ctrl | 22 | Acute myocardial infarct | I |
| 54 | C | M | Ctrl | 17 | Atherosclerotic cardiovascular disease | I |
| 55 | - | F | Ctrl | 12 | Cardiomegaly | I |
| 56 | C | M | Ctrl | 19 | Gunshot wound to chest, homicide | I |
| 57 | C | M | Ctrl | 13 | Atherosclerotic cardiovascular disease | I |
| 63 | AA | M | Ctrl | 20 | Pulmonary embolism | I |
| 64 | C | F | Ctrl | 20 | Atherosclerotic cardiovascular disease | I |
| 66 | C | F | Ctrl | 25 | Subarachnoid hemorrhage | E |
| 68 | C | M | Ctrl | 13 | Atherosclerotic cardiovascular disease | I |
| 70 | AA | M | Ctrl | 12 | Atherosclerotic cardiovascular disease | I |
| 73 | C | F | Ctrl | 13 | Peritonitis | I |
| 76 | C | M | Ctrl | 3 | Atherosclerotic cardiovascular disease | I |
| 82 | C | M | Ctrl | 13 | Atherosclerotic cardiovascular disease | I |
| 93 | C | F | Ctrl | 14 | Tau astrogliopathy | E |
| 35 | C | F | SZ | 7 | Substance overdose | I |
| 39 | C | M | SZ | 16 | Substance overdose | I |
| 40 | AA | F | SZ | 11 | Cardiac arrhythmia | I |
| 41 | AA | F | SZ | 7 | Pneumonia | I |
| 42 | C | M | SZ | 6 | Atherosclerotic cardiovascular disease | I |
| 47 | C | F | SZ | 22 | Asphyxia | I |
| 48 | C | F | SZ | 7 | Pulmonary thromboembolism | I |
| 48 | AA | M | SZ | 19 | Atherosclerotic cardiovascular disease | I |
| 48 | AA | F | SZ | 6 | Asphyxia | I |
| 50 | AA | M | SZ | 13 | Sudden death associated with schizophrenia | I |
| 50 | C | M | SZ | 24 | Atherosclerotic cardiovascular disease | I |
| 53 | C | M | SZ | 14 | Atherosclerotic cardiovascular disease | I |
| 54 | C | M | SZ | 19 | Atherosclerotic cardiovascular disease | I |
| 56 | C | F | SZ | 20 | Atherosclerotic cardiovascular disease | I |
| 57 | C | M | SZ | 16 | Myocardial infarction | I |
| 57 | C | M | SZ | 11 | Allergic reaction | I |
| 62 | C | M | SZ | 8 | Chronic obstructive pulmonary disease | I |
| 66 | AA | F | SZ | 5 | Atherosclerotic cardiovascular disease | I |
| 67 | C | F | SZ | 4 | Atherosclerotic cardiovascular disease | I |
| 68 | AA | M | SZ |  | Undetermined | I |
| 70 | AA | M | SZ | 20 | Atherosclerotic cardiovascular disease | I |
| 71 | C | F | SZ | 5 | Atherosclerotic cardiovascular disease | I |
| 76 | C | M | SZ | 7 | Atherosclerotic cardiovascular disease | I |
| 84 | C | M | SZ | 5 | Atherosclerotic cardiovascular disease | I |
| 93 | C | F | SZ | 14 | Complications of disorder | I |
| PMI = Postmortem Interval, reported in hours; AA = African American; C = Caucasian; Ctrl = control; SZ = schizophrenia; I/E = inclusion/exclusion | | | | | | |
